# Traceability of Satsuma Mandarin (*Citrus unshiu* Marc.) Honey through Nectar/Honey-Sac/Honey Pathways of the Headspace, Volatiles, and Semi-Volatiles: Chemical Markers

**DOI:** 10.3390/molecules21101302

**Published:** 2016-09-29

**Authors:** Igor Jerković, Saša Prđun, Zvonimir Marijanović, Marina Zekić, Dragan Bubalo, Lidija Svečnjak, Carlo I. G. Tuberoso

**Affiliations:** 1 Department of Organic Chemistry, Faculty of Chemistry & Technology, University of Split, Ruđera Boškovića 35, HR-21000 Split, Croatia; zekic@ktf-split.hr; 2 Department of Fisheries, Apiculture and Special Zoology, Faculty of Agriculture, University of Zagreb, Svetošimunska 25, HR-10000 Zagreb, Croatia; sprdjun@agr.hr (S.P.); dbubalo@agr.hr (D.B.); lsvecnjak@agr.hr (L.S.); 3 Department of Food Technology, Marko Marulić Polytechnic in Knin, Petra Krešimira IV 30, HR-22300 Knin, Croatia; zmarijanovic@veleknin.hr; 4 Department of Life and Environmental Sciences, University of Cagliari, via Ospedale 72, IT-09124 Cagliari, Italy; tuberoso@unica.it

**Keywords:** *Citrus unshiu* Marc. honey, phenylacetaldehyde, phenylacetonitrile, linalool and its derivatives, 1*H*-indole, 1,3-dihydro-2*H*-indol-2-one, caffeine, HS-SPME, USE, GC-MS

## Abstract

Headspace solid-phase microextraction (HS-SPME) and ultrasonic solvent extraction (USE), followed by GC-MS/FID, were applied for monitoring the nectar (NE)/honey-sac (HoS)/honey (HO) pathways of the headspace, volatiles, and semi-volatiles. The major NE (4 varieties of *Citrus unshiu*) headspace compounds were linalool, α-terpineol, 1*H*-indole, methyl anthranilate, and phenylacetonitrile. Corresponding extracts contained, among others, 1*H*-indole, methyl anthranilate, 1,3-dihydro-2*H*-indol-2-one and caffeine. The major HoS headspace compounds were linalool, α-terpineol, 1,8-cineole, 1*H*-indole, methyl anthranilate, and *cis*-jasmone. Characteristic compounds from HoS extract were caffeine, 1*H*-indole, 1,3-dihydro-2*H*-indol-2-one, methyl anthranilate, and phenylacetonitrile. However, HO headspace composition was significantly different in comparison to NE and HoS with respect to phenylacetaldehyde and linalool derivatives abundance that appeared as the consequence of the hive conditions and the bee enzyme activity. *C. unshiu* honey traceability is determined by chemical markers: phenylacetaldehyde, phenylacetonitrile, linalool and its derivatives, as well as 1*H*-indole, 1,3-dihydro-2*H*-indol-2-one, and caffeine.

## 1. Introduction

The most common and economically important varieties of Satsuma mandarin (*Citrus unshiu* Marc.) in Croatia (Neretva valley, Opuzen area) are *Wakiyama*, *Chahara*, *Okitsu*, *Kawano Wase*, *Owari*, *Saigon*, *Kuno*, *Zorica*, *Ichumare*, and *Seto* [1]. Around 2.5 million mandarin trees have been planted [1] in the Opuzen area (ca. 2500 ha) providing a good nectar source for unifloral honey production and potential for independent commercialization (not just as *Citrus* honey without distinction of the species). *C. unshiu* honey has not yet been characterized in detail.

Melissopalynological analysis, based on the identification and quantification of the pollen percentage by microscopic examination, has been accepted as the reference method to authenticate honey botanical origin [2]. However, pollen analysis is considered of little value for the *Citrus* genus as it is one of several honey types with underrepresented pollen [3,4]. Accordingly, *C. unshiu* honey characterisation is difficult with underrepresented pollen due to the specific plant physiology of particular mandarin cultivars (aborted anthers, sterile pollen grains, or partenocarpy). Therefore, there is a need for detailed chemical characterisation of *C. unshiu* honey and present research is focused on its volatile organic compounds (VOCs). The usefulness of VOC analysis in the determination of the honey botanical origin has been reported previously [5,6,7]. The aroma profile can be considered to be a “fingerprint” of unifloral honey directly related to the plant nectar [8].

*Citrus* honey has been often analysed without distinction between different species (*C. sinensis* L., *C. deliciosa* Ten., *C. limon* L., etc.) or, at best, as orange/lemon blossom honey. The extracts from *Citrus* honey contain linalool derivatives, such as (*E*)-2,6-dimethylocta-2,7-diene-1,6-diol, 2,6-dimethylocta-3,7-diene-2,6-diol or (*Z*)-2,6-dimethylocta-2,7-diene-1,6-diol [9]. Higher concentrations of linalool oxides and lilac aldehydes/alcohols were also found in the honey headspace [10,11,12,13]. α-4-Dimethylcyclohex-3-ene-1-acetaldehyde was present in Greek *Citrus* honey [10]. Methyl anthranilate has been suggested as a *Citrus* honey floral marker [14]. Two isomers of sinensal, the volatile component of orange essential oil, were identified in Spanish *Citrus* honey [12]. The enantiomeric ratio between (3*S*)-linalool and (3*R*)-linalool in orange honey was about 13:87 similar as in orange flowers, and *trans*-(2*R*,5*R*)-linalool oxide with *cis*-(2*S*,5*R*)-linalool oxide prevailed [15]. The volatiles composition, physicochemical parameters, flavonoids and phenolic compounds were used as tools in the statistical analysis for the differentiation of lemon and orange honey [16,17]. A stepwise discriminant analysis using 37 volatiles and different parameters (diastase, conductivity, Pfund colour, and CIE L*a*b*) provided the model for classification of the samples. A multivariate PLS2 analysis showed that lilac aldehydes and phenylacetaldehyde (all abundant in orange honey) were negatively correlated with four flavonoids (pinocembrin, chrysin, naringenin, and quercetin) and caffeic acid, all abundant in lemon honey. Moreover, the last five compounds were positively correlated with six alcohols, two ketones, acetaldehyde, and furanmethanol. In addition, caffeine is present in *Citrus* honey at about 1–10 mg/kg [18].

Basic physicochemical parameters of *C. unshiu* honey were determined in our previous research [19] with the following average values: water content 16.00%, electrical conductivity 0.23 mS·cm^−1^, fructose and glucose 77.33%, saccharose 1.5%, free acidity 15.10 mEq·kg^−1^ and diastase activity 10.60 (DN). The present research emphases: (a) the headspace, volatile and semi-volatile nectar compounds from the varieties *Zorica*, *Chahara*, *Kawano Wase*, and *Okitsu*, from the honey-sac of the bees returning from *C. unshiu* pastures and corresponding honey; (b) GC-FID and GC-MS analysis of the headspace using headspace solid-phase microextraction (HS-SPME) and the volatiles and semi-volatiles after ultrasonic solvent extraction (USE); (c) methyl anthranilate, caffeine, and other compounds’ abundances in the nectar/honey-sac/honey with respect to *C. unshiu* honey traceability; (d) the comparison of applied extraction methods with respect to obtained chemical profiles; and (e) qualitative comparison with available data on other *Citrus* honey VOCs.

## 2. Results and Discussion

This research has been designed to follow the nectar (NE)/honey-sac (HoS)/honey (HO) pathways of the headspace, volatiles, and semi-volatiles in order to characterize Satsuma mandarin (*Citrus unshiu* Marc.) honey. Two complementary methods for VOC isolation (without artefact generation) were applied: HS-SPME (with two fibres: divinylbenzene/carboxene/polydimethylsiloxane (DVB/CAR/PDMS) and polydimethylsiloxane/divinylbenzene (PDMS/DVB)) and USE (using two solvents: pentane/Et_2_O 1:2 (*v*/*v*) and CH_2_Cl_2_). The applied chemical screening methodologies indicated striking differences in the obtained chemical profiles (depending on the volatility/solubility/sample). As was previously mentioned, the melissopalynological analysis cannot be used ambiguously to authenticate this honey. To ensure the unifloral honey origin, all of the samples were collected under controlled bee-hive locations in the area where *C. unshiu* predominantly grows. In investigated samples of the local beekeepers Satsuma mandarin pollen ranged from 1%–10%, and in other samples 3%–41%. The pollen grains from other nectar plant species in the samples were: *Capsela bursa pastoris* L., Asteraceae (*Taraxacum* form), *Citrus sinensis* L., *Fabaceae* spp., *Rhamnus* spp., *Colutea arborescens* L., *Diplotaxis erucoides* L., *Centaurea* spp. and *Cerastium* spp.

### 2.1. The Nectars’ Chemical Composition of the Headspace, Volatiles and Semi-Volatiles

**Figure 1:**
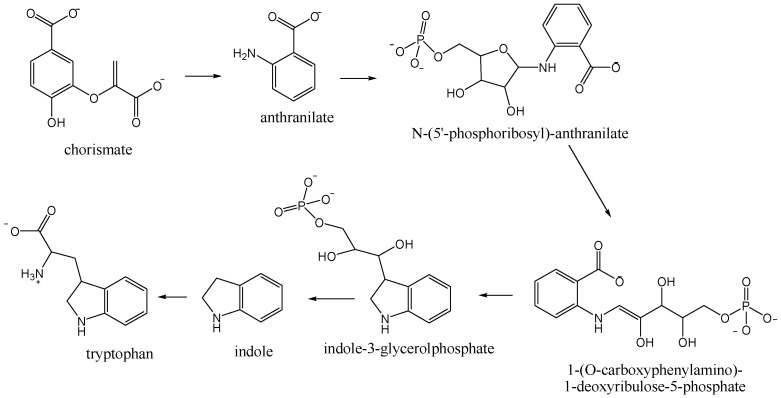
Tryptophan biosynthetic pathway.

The major headspace compounds from all nectar varieties were nitrogen-containing compounds 1*H*-indole (7.3%–52.5%; 12.2%–47.4%) and methyl anthranilate (3.0%–8.5%; 5.6%–19.8%), Table 1. Higher percentages of 1*H*-indole was found in NE *Okithu* and *Zorica* and methyl anthranilate in NE *Kawano Wase*. Those compounds derive from chorismate in the tryptophan biosynthetic pathway (Figure 1). The plant enzymes catalyse three subsequent steps [20]. PR-anthranilate transferase catalyses phosphoribosyl moiety transfer from phospho-ribosylpyrophosphate to anthranilate. In the next step, PR-anthranilate isomerase rearranges PR-anthranilate to 1-(*O*-carboxyphenylamino)-1-deoxyribulose-5-phosphate. Indole-3-glycerolphosphate synthase next forms an indole ring during the conversion of 1-(*O*-carboxyphenyIamino)-1-deoxyribulose-5-phosphate to indole-3-glycerolphosphate.

The major oxygenated monoterpenes in NE headspace were linalool (1.2%–21.7%; 5.3%–21.1%) and α-terpineol (2.4%–16.0%; 4.1%–9.3%). Their highest percentages were found in NE *Kawano Wase* and *Chahara*. Other abundant monoterpenes were terpinen-4-ol (0.0%–5.9%; 0.7%–3.6%), 1,8-cineole (0.4%–2.8%; 0.0%–3.7%) and γ-terpinene (0.0%–4.4%; 0.0%–8.1%). All these compounds are biosynthetically related and derive from geranyl pyrophosphate. Other monoterpenes were occasionally found in several nectar types (Table 1). Only a few sesquiterpenes, pharnesyl pyrophosphate derivatives, were present with *trans*-caryophyllene (0.0%–2.2%; 0.0%–2.6%) as the major contributor.

**Figure 2:**
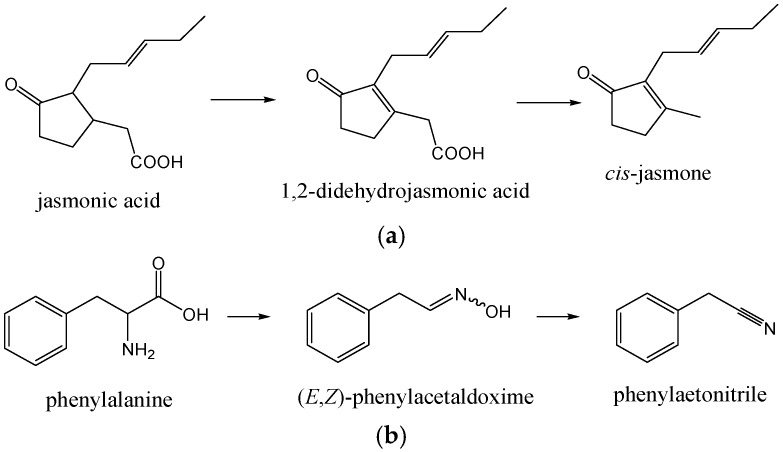
(**a**) Formation of *cis*-jasmone.; and (**b**) formation of phenylacetonitrile.

*cis*-Jasmone (*cis*-3-methyl-2-(2-pentenyl)-cyclopent-2-en-1-one) was found (1.1%–3.6%; 2.8%–7.9%) in all NE. It is produced by the plants by an oxidative degradation of jasmonic acid (formed by lipoxygenase-catalyzed oxygenation of linolenic acid via 18-carbon cyclic fatty acid formed by the action of hydroperoxide cyclase, followed by reduction and β-oxidations), via 1,2-didehydrojasmonic acid [21]. Subsequent protonation of the carbonyl *O*-atom of 1,2-didehydrojasmonic acid is assumed to induce a Grob-type fragmentation of the molecule, yielding CO_2_ and *cis*-jasmone (Figure 2a).

Among benzene derivatives formed by the shikimate biosynthetic pathway, 2-phenylethanol (1.3%–4.1%; 0.6%–2.8%) and benzaldehyde (0.1%–3.6%; 0.0%–0.6%) were abundant with different distributions among NE_A_–NE_D_ (Table 1). Benzyl alcohol, phenylacetaldehyde (more abundant in NE *Okitsu* and *Zorica*) and methyl benzoate were also found. Phenylacetonitrile (0.0%–7.3%; 0.0%–6.3%) is another benzene derivative containing nitrogen. Its formation has been found in several secondary metabolic pathways initiating from phenylalanine in the plants [22]. Phenylalanine is first converted to (*E*,*Z*)-phenylacetaldoxime, which is then transformed to 2-hydroxy-2-phenylacetonitrile, probably via phenylacetonitrile formation as the intermediate (Figure 2b).

NE_A_–NE_D_ headspace also contained lower aliphatic compounds up to C_10_ (Table 1), most probably derived from fatty acid degradation. These compounds include alcohols (e.g., (*Z*)-hex-3-en-1-ol, pentan-1-ol or hexan-1-ol), ketones (e.g., heptan-2-one or octan-2-one), acids (acetic and hexanoic), and methyl esters (octanoate, nonanoate and decanoate).

Ultrasonic solvent extracts of NE_A_–NE_D_ were strikingly different from the headspace composition, containing less volatile compounds (Table 2). The major ones were (*Z*)-octadec-9-en-1-ol (14.3%–52.3%), octadecan-1-ol (5.3%–26.0%) and hexadecan-1-ol (6.9%–16.5%). The headspace nitrogen-containing compounds were also identified by USE: 1*H*-indole (2.7%–10.3%) and methyl anthranilate (0.9%–11.7%). However, new nitrogen compound appeared in the extracts, 1*H*-indole derivative—1,3-dihydro-2*H*-indol-2-one (0.2%–18.1%). It is interesting to note that the Schiff base of 1,3-dihydro-2*H*-indol-2-one and its synthetic derivatives bearing a 1,3-dihydro-2*H*-indol-2-one nucleus were reported for antibacterial, antifungal, anti-HIV, and anticonvulsant activities and GAL3 receptor antagonists [23].

Methylxanthine-derivative caffeine was found (1.1%–8.9%) in the extracts of NE_A_–NE_D_. Xanthine (3,7-dihydro-1*H*-purine-2,6-dione) derivatives belong to purine alkaloids. The major pathway of caffeine biosynthesis is xanthosine → 7-methylxanthosine → 7-methylxanthine → theobromine → caffeine [24]. The presence of methyxanthine derivatives in the nectar and pollen of *Coffea*, *Camellia*, *Theobroma*, *Herrania*, *Cola*, *Ilex*, *Paullinia*, and *Citrus* spp. was previously determined by HPLC [18]. The nectar of *C. paradise*, *C. maxima*, and *C. limon* contained caffeine (60–487 nmol·mL^−1^), theobromine (0–22 nmol·mL^−1^), theophylline (0–55 nmol·mL^−1^) and paraxanthine (0–12 nmol·mL^−1^). It is difficult to compare the abundance of caffeine (Table 2) determined by USE/GC-MS/FID with the results of direct HPLC analysis, which is focused on the non-volatiles analysis. 

Generally, Satsuma mandarin peel essential oil was characterized by a high percentage of limonene (67.4%), followed by β-myrcene, car-3-ene, α-pinene, *p*-cymene, β-pinene, sabinene, terpinolene, and α-thujene [25]. The principal chemical constituents of *C. unshiu* flower essential oil included γ-terpinene (24.7%), β-pinene (16.6%), *o*-cymene (11.5%), limonene (5.7%), β-ocimene (5.6%) and α-pinene (4.7%) [25]. It is already known that the composition of the nectar and essential oil from the same plant is very different [26]. However, several common compounds were found in the flower essential oil and *C. unshiu* nectar headspace investigated herein, such as γ-terpinene, limonene, α-pinene, and β-pinene.

### 2.2. The Chemical Composition of the Content of Honey-Sac Headspace, Volatiles and Semi-Volatiles

The gathered nectar is stocked in the honey-sac, which can contain up to 60 μL. The enzymes in the saliva start to degrade the nectar sucrose into glucose and fructose and cleave the glycosides. The content of the sacs of the bees caught at the entrance of the hive on their way back from *C. unshiu* nectar-gathering was investigated by HS-SPME/GC-MS/FID (Table 3) and USE/GC-MS/FID (Table 4). Dominant compounds were oxygenated monoterpenes, the major ones were linalool (15.2%; 3.8%), α-terpineol (10.2%; 9.3%) and 1,8-cineole (1.9%; 3.6%), Table 3. Two nitrogen-containing compounds were found among the abundant compounds: 1*H*-indole (7.9%; 8.9%) and methyl anthranilate (7.7%; 19.8%). *cis*-Jasmone was also present (5.1%; 6.9%). The headspace benzene derivatives were mainly comprised of benzaldehyde (1.0%; 2.7%), benzyl alcohol (2.9%; 3.6%), phenylacetaldehyde (0.5%; 2.0%) and phenylacetonitrile (11.0%; 10.4%). The comparison with the headspace of all nectars reveals dominant qualitative similarities regarding the major compounds with fluctuation among their percentages (Table 1). Two compounds were found only in the HoS headspace, not in the nectars: pentan-1-ol (1.3%; 1.3%) and heptan-2-ol (7.8%; 3.3%). 

USE extract of HoS contained higher aliphatic compounds, the major ones were (*Z*)-octadec-9-en-1-ol (45.3%), octadecan-1-ol (8.6%), and hexadecan-1-ol (11.6%), Table 4. These chemical structures are related to the composition of cuticular waxes and less to pheromones, but have been also found in NE (Table 2). Fatty acids and alcohols were previously identified as the major compounds of the solvent organic extract from the sacs of the bees that collected *Mentha* spp. nectar, and methyl syringate, terpendiol I and vomifoliol were attributed to the plant origin [27]. Other important compounds from Table 2 were caffeine (11.5%), 1H-indole (1.6%), 1,3-dihydro-2*H*-indol-2-one (6.4%), and methyl anthranilate (1.4%). USE extracts of HoS and NE were very similar. Only 1-hydroxylinalool appeared (5.4%) in HoS, which can be indication of the beginning of linalool transformations triggered by the enzymes and continued later in the combs.

### 2.3. The Chemical Composition of C. unshiu Honey Headspace, Volatiles and Semi-Volatiles

On returning to the hive, the content of the honey-sac is regurgitated into the honeycomb and ripened into honey. Under the honeycomb oxidative atmosphere sensitive honey organic compounds can undergo oxidation [28]. There are only a few studies in which the organic extractives of the honey-sac have been correlated with those of the corresponding honey. The comparison of the components of the extracts of Linden honey and honey-sac contents showed that nectar and honey-sac contents contain many aldehydes which were found as corresponding acids in the honey, while the aliphatic compounds, isoprenoids and the alkaloids remained unchanged [28]. In another research, the major identified terpene in the honey-sac was 3,7-dimethylocta-1,5-dien-3,7-diol (terpendiol I) and it was found in *Mentha* spp. honey solvent extracts, but also can transform to hotrienol, the most abundant compound in the honey headspace [27].

Phenylacetaldehyde was dominant compound (34.4%–47.2%; 38.3%–49.1%) of the *C. unshiu* honey headspace, followed by benzaldehyde (5.8%–9.8%%; 3.3%–6.6%). Among other benzene derivatives, abundant was phenylacetonitrile (2.7%–9.9%; 3.4%–10.2%). Phenylacetaldehyde was strikingly more abundant in comparison with the nectar headspace (HS-NE) and the headspace of the honey-sac (HS-HoS), shown in Table 1, indicating its formation during the honey ripening in the hive, since heat was not applied to the samples. This can be generated from phenylalanine either by enzyme catalysis or by Strecker degradation [29]. A high percentage of phenylacetaldehyde was found in the honey headspace of *Asphodelus microcarpus* Salz. et Viv. [30]. Phenylacetonitrile was present within percentage ranges similar to those seen in the HS-NE and HS-HoS (Table 1), while benzaldehyde percentages were elevated. In addition to phenylacetonitrile, two aliphatic nitriles were detected in several honey samples with minor percentages (Table 3): ethylisocyanide and 3-methylbutanenitrile. Benzaldehyde was found to be the major volatile from the honey of cambara and willow, but also in lemon and orange honey [17,29]. Phenylacetonitrile was found in the headspace of dandelion and thyme honeys [31,32].

Linalool was present as a minor constituent (0.0%–2.2%; 0.0%–4.5%) in distinction to HS-NE and HS-HoS. However, an array of linalool derivatives were found, such as *cis*-linalool oxide (3.0%–11.5%; 0.0%–4.1%), hotrienol (1.4%–2.6%; 1.2%–2.3%), lilac aldehydes, dill ether or *p*-menth-9-en-1-al isomers, not present at all in HS-NE and HS-HoS. They were formed from linalool within the hive conditions. Hotrienol derive either from dehydration of 2,6-dimethylocta-3,7-diene-2,6-diol or from allylic rearrangement and dehydration of 3,7-dimethylocta-1,7-diene-3,6-diol, which can be liberated from the corresponding glucoside or from dehydration of 8-hydroxylinalool [7]. Lilac aldehydes are formed by oxidation of lilac alcohols generated by hydroxylation of linalool to (*E*)-8-hydroxylinalool, and further to (*E*)-8-oxolinalool. Dill ether and *p*-menth-1-ene-9-al isomers and *p*-menth-1-ene-8-ol (α-terpineol) are also derived from (*E*)-8-hydroxylinalool via allylic rearrangement and cyclisation of 8-hydroxygeraniol. Epoxidation of linalool gives 6,7-epoxylinalool, which undergoes further reactions to form linalool oxides and anhydrolinalool oxides, which can further yield lilac alcohols. The formation of linalool oxides and 2,6-dimethylocta-3,7-diene-2,6-diol was probably catalysed by the enzymes secreted by the bees [7].

1*H*-indole and methyl anthranilate were occasionally present, but not in the headspace of all honey samples, and with markedly lower percentages in comparison to HS-NE and HS-HoS.

Among lower aliphatic compounds of the honey headspace, nonanoic acid was the most abundant (2.2%–3.3%; 2.3%–4.9%), but not found in HS-NE and HS-HoS. 

Predominant compounds of the extracts were higher aliphatic compounds, such as (*Z*)-octadec-9-en-1-ol (20.5%–49.1%; 11.8%–22.8%), hexadecanoic acid (0.5%–6.6%; 7.3%–33.8%), octadecan-1-ol (4.5%–11.4%; 3.8%–5.4%) and hexadecan-1-ol (5.8%–15.3%; 2.4%–5.4%). These compounds (except hexadecanoic acid) were found also in the nectar extracts (E-NE) and the honey-sac extract (E-HoS), but cannot be connected with specific botanical origin since they can be transferred from the comb environment [33]. Similar applies for higher alkanes found only in the honey extracts: tetracosane (1.1%–20.8%; 0.1%–29.7%), tricosane (0.3%–26.4%), docosane (0.2%–15.7%; 0.0%–8.5%) or heneicosane (0.0%–3.7%; 0.0%–3.0%). Among interesting compounds of the extracts, 1*H*-indole (0.1%–0.3%; 0.1%–2.6%) and 1,3-dihydro-2*H*-indol-2-one (0.2%–1.7%; 0.1%–1.3%) can be pointed out, similar to E-NE and E-HoS. Caffeine was also detected in all of the extracts (0.3%–0.8%; 1.2%–7.1%) with lower abundance in comparison to E-NE and E-HoS. In distinction to E-NE and E-HoS, two interesting compounds were present in the honey extracts: 1-hydroxylinalool (0.5%–6.5%; 0.2%–2.0%) and vomifoliol (0.2%–2.0%; 0.2%–2.4%). 1-Hydroxylinalool, previously identified in E-HoS, is a good indicator of linalool transformations providing linalool derivatives found only in the honey headspace. Norisoprenoid vomifoliol was not found in E-NE and E-HoS and, therefore, could be transferred from the comb environment. Pinocembrin (5,7-dihydroxy-2-phenyl-2,3-dihydro-4*H*-chromen-4-one) was found only in CH_2_Cl_2_ extracts. It is a flavanone, containing the nucleus of hesperetin (2,3-dihydro-5,7-dihydroxy-2-(3-hydroxy-4-methoxyphenyl)-4*H*-1-benzopyran-4-one) that was found in *Citrus* honeys [13]. Due to the high molecular mass of pinocembrin, GC-MS is not a good method for its quantification (as for other flavanones). In addition, it could originate from propolis and, therefore, it is not commented in detail. 

In comparison with other *Citrus* honey VOCs, several similarities can be pointed out. Namely, the suitability of methyl anthranilate, originating from the plant, as the chemical marker of *Citrus* honey types has been already found [34]. Along with the detection of methyl anthranilate, more than 60 different VOCs were also reported [11,12,17] in *Citrus* honey types. Similar to the present research, benzaldehyde, phenylacetaldehyde, and linalool derivatives (e.g., linalool oxides, lilac aldehyde isomers, or *p*-menth-1-en-9-al) were found among important *Citrus* honey headspace compounds. Caffeine was also previously found as a characteristic compound of *Citrus* honey [35]. However, despite previous studies on *Citrus* honeys, few particular compounds were present in *C. unshiu* honey not already mentioned in other *Citrus* honeys, such as 1*H*-indole, 1,3-dihydro-2*H*-indol-2-one, and phenylacetonitrile.

## 3. Materials and Methods

### 3.1. The Nectar, Honey-Sac and Honey Samples

The nectars (1.5 mL) from the varieties *Zorica*, *Chahara*, *Kawano Wase*, and *Okitsu* were collected with microcapillary glass tubes from trees growing in the Neretva valley, Opuzen area, Croatia, in 2016. In the study area 90% of *Citrus* orchards were Satsuma mandarins (*Citrus unshiu* Marc.), while others were clementine (*C. clementina* Hort. ex Tan.), sweet orange (*C. sinensis*), grapefruit (*C. paradisi*), and lemon (*C. limon*).

During *C. unshiu* honey flow, a part of the returning foragers were collected. The bees were frozen in the field by liquid nitrogen and were stored in a deep-freezer until their honey-sac contents were investigated. After thawing, the abdomen of 100 bees was dissected by peeling off the tergit with forceps in order to expose the honey sac. The honey sacs were removed and frozen. After freezing, the entire content of the honey-sacs was pooled and put in a glass vial (5 mL) at 4 °C until the volatiles were isolated.

Twelve *C. unshiu* honey samples were investigated. The combs from specially prepared colonies, which were formed from 2 kg of packaged bees on wax foundation, were placed in the area of predominantly *C. unshiu* trees growing in the Neretva valley, Opuzen area, Croatia, but the samples were also collected from local beekeepers. All of the samples were stored in hermetically closed glass bottles at 4 °C until the volatiles were isolated. Melissopalynological analysis was performed by the method recommended by the International Commission for Bee Botany [36]. Microscopical examination was carried out on a Hund h 500 (Wetzlar, Germany) light microscope attached to a digital camera (Motic m 1000, Motic Deutschland GmbH, Wetzlar, Germany) and coupled to an image analysis system (Motic Images Plus software, Motic Deutschland GmbH) for morphometry of pollen grains. 

### 3.2. Headspace Solid-Phase Microextraction (HS-SPME)

The headspace extraction was performed using a manual SPME holder using two fibres: divinylbenzene/carboxene/polydimethylsiloxane (DVB/CAR/PDMS) and polydimethylsiloxane/divinylbenzene (PDMS/DVB) obtained from Supelco Co. (Bellefonte, PA, USA). The fibres were conditioned prior to use according to the instructions by Supelco Co. For HS-SPME, the nectars (1 mL) were placed separately in 5 mL glass vials and hermetically sealed with PTFE/silicone septa. The content of honey-sacs was put as described above in 5 mL glass vial and hermetically closed with PTFE/silicone septa. The honey/saturated water solution (5 mL, 1:1 (*v*/*v*); saturated with NaCl) of each honey sample was placed in a 15 mL glass vial and hermetically sealed with PTFE/silicone septa.

The vials were maintained in a water bath at 60 °C during equilibration (15 min) and HS-SPME (45 min) and were partially submerged so that the liquid phase of the sample was below the water level. All of the experiments were performed under constant stirring (1000 rpm) with a magnetic stirrer. After sampling, the SPME fibre was withdrawn into the needle, removed from the vial, and inserted into the injector (250 °C) of the GC-FID and GC-MS for 6 min where the extracted volatiles were thermally desorbed directly to the GC column.

### 3.3. Ultrasonic Solvent Extraction (USE)

Ultrasound-assisted solvent extraction (USE) was performed in an ultrasound cleaning bath (Clean 01, MRC Scientific Instruments, London, UK) by the indirect sonication mode at a frequency of 37 kHz at 25 ± 3 °C. Two solvents were separately used for USE: a mixture of pentane/diethyl ether, 1:2 (*v*/*v*) and dichloromethane.

The nectars (0.5 mL) were separately dissolved in flasks (5 mL) in 0.5 mL distilled water, MgSO_4_ (0.05 g) was added and the sample was vortexed (5 min). Dichloromethane (1 mL) was used for USE of dissolved nectars. 

The content of honey-sacs was dissolved in distilled water (0.5 mL) in 5 mL flask, MgSO_4_ (0.03 mg) was added, and the sample was vortexed (5 min). USE was performed using dichloromethane (1.5 mL). Forty grams of each *C. unshiu* honey sample was dissolved in distilled water (22 mL) in a 100-mL flask. Magnesium sulphate (1.5 g) was added and each sample was vortexed (10 min). Both solvents (20 mL) were separately used for USE of the honey samples. 

The sonication was maintained for 30 min. After sonication, the organic layer was separated by centrifugation and filtered over anhydrous MgSO_4_. The aqueous layer was returned to the flask and another batch of the same extraction solvent was added and extracted by ultrasound for 30 min. The organic layer was separated in the same way as the previous one and filtered over anhydrous MgSO_4_, and the aqueous layer was sonicated a third time for 30 min with another batch of the extraction solvent. Combined organic extracts were concentrated to 0.2 mL by distillation with a Vigreaux column, and 1 μL was used for GC-FID and GC-MS analyses.

### 3.4. GC-FID and GC-MS Analyses

The GC-FID analyses were carried out with an Agilent Technologies (Palo Alto, CA, USA) gas chromatograph model 7890A equipped with a flame ionization detector (FID) and a HP-5MS capillary column (5% phenyl-methylpolysiloxane, Agilent J and W). The GC conditions were similar to those described previously [24]. In brief, the oven temp. was programmed isothermal at 70 °C for 2 min, increasing from 70–200 °C at 3 °C·min^−1^, and held isothermally at 200° for 15 min; carrier gas, He (1.0 mL·min^−1^).

The GC-MS analyses were performed using an Agilent Technologies (Palo Alto, CA, USA) gas chromatograph model 7820A equipped with a mass selective detector (MSD) model 5977E (Agilent Technologies) and a HP-5MS capillary column, under the same conditions as described for the GC-FID analysis. The MSD (EI mode) was operated at 70 eV, and the mass range was 30–300 amu, as previously reported [24].

The identification of the volatile constituents was based on the comparison of their retention indices (RI), determined relative to the retention times of a homologous series of *n*-alkanes (C_9_-C_25_), with those reported in the literature [25] and their mass spectra with authentic compounds available in our laboratories or those listed in Wiley 9 (Wiley, New York, NY, USA) and NIST 14 (D-Gaithersburg) mass spectral libraries [25]. The percentage composition of the samples was computed from the GC peak areas using the normalization method (without correction factors). The average component percentages in the tables were calculated from duplicate GC-FID and GC-MS analyses.

## 4. Conclusions

Applied HS-SPME/GC-MS/FID and USE/GC-MS/FID methodologies of monitoring nectar/honey-sac/honey pathways of the headspace, volatiles, and semi-volatiles was successful and complementary for the characterisation of *C. unshiu* honey. The major headspace compounds from all nectar varieties were linalool, α-terpineol, 1*H*-indole, methyl anthranilate, and phenylacetonitrile. Corresponding extracts contained, among others, 1*H*-indole, methyl anthranilate, 1,3-dihydro-2*H*-indol-2-one and caffeine. The major headspace compounds of the honey-sac were linalool, α-terpineol, 1,8-cineole, 1*H*-indole, methyl anthranilate, and *cis*-jasmone. Characteristic compounds from related extract were caffeine, 1*H*-indole, 1,3-dihydro-2*H*-indol-2-one, methyl anthranilate, and phenylacetaldehyde. However, the honey headspace composition was significantly different in comparison to the nectars and the honey-sac content with respect to phenylacetaldehyde and linalool derivatives’ abundances that appeared as the consequence of the hive conditions and the bee enzymes’ activity. All extracts contained higher aliphatic compounds as the major constituents not useful for botanical origin determination, since they can originate from the comb environment. *C. unshiu* honey traceability is determined by the following chemical markers: phenylacetaldehyde, phenylacetonitrile, linalool, and its derivatives (from the headspace), as well as 1*H*-indole, 1,3-dihydro-2*H*-indol-2-one, and caffeine (from the extracts). 1*H*-Indole, 1,3-dihydro-2*H*-indol-2-one, and phenylacetonitrile were found as particular compounds of *C. unshiu* honey, not pointed out in previous studies of other *Citrus* honey types.

## Figures and Tables

**Table 1:** The headspace chemical composition of the honey-sac (HoS) and nectars (NE) of different *C. unshiu* varieties determined by HS-SPME/GC-MS/FID analysis.

No.	Compound	RI	Area (%) Fibre PDMS/DVB					Area (%) Fibre DVB/CAR/PDMS				
			HoS	NE_A_	NE_B_	NE_C_	NE_D_	HoS	NE_A_	NE_B_	NE_C_	NE_D_
1	Acetic acid	<900	1.8	0.4	0.3	-	-	4.9	-	-	-	0.9
2	3-Hydroxybutan-2-one	<900	-	0.2	0.3	-	-	-	-	-	-	-
3	Pentan-1-ol	<900	1.3	-	-	-	-	1.3	-	-	-	-
4	Hexanal	<900	-	0.1	0.1	0.5	0.7	-	-	-	0.3	-
5	(*Z*)-Hex-3-en-1-ol	<900	-	2.9	1.7	0.2	-	-	0.3	0.1	-	-
6	Hexan-1-ol	<900	-	4.9	5.2	-	-	-	0.5	0.3	-	-
7	Heptan-2-one	<900	5.5	0.7	-	-	-	-	0.2	-	-	-
8	Heptan-2-ol	<900	7.8	-	-	-	-	3.3	-	-	-	-
9	α-Pinene	940	-	0.1	0.1	-	-	-	0.1	0.3	-	-
10	Benzaldehyde	965	1.0	0.1	0.3	2.2	3.6	2.7	-	-	0.6	-
11	Hexanoic acid	980	-	-	-	-	-	0.9	-	0.5	-	-
12	β-Pinene	982	-	0.1	0.1	-	0.4	-	0.5	-	-	-
13	6-Methylhept-5-en-2-one	989	-	0.6	0.8	0.2	-	-	0.5	0.3	0.6	-
14	Octan-2-one	993	-	1.1	0.4	-	-	-	1.5	-	-	-
15	β-Myrcene	994	-	-	-	-	-	-	-	0.4	-	-
16	6-Methylhept-5-en-2-ol	995	-	-	0.4	-	-	-	-	0.0	-	-
17	α-Terpinene	1022	-	0.1	0.1	-	-	-	-	0.4	-	-
18	*p*-Cymene	1029	4.0	3.0	2.1	-	1.1	-	3.6	3.7	0.3	-
19	Limonene	1034	-	1.3	0.9	-	-	-	1.0	1.5	-	-
20	1,8-Cineole	1037	1.9	2.8	1.3	0.3	0.4	3.6	3.1	1.5	0.6	-
21	Benzyl alcohol	1038	2.9	-	-	-	-	3.6	-	-	-	-
22	*cis*-β-Ocymene	1042	-	-	-	-	-	-	0.2	0.1	-	-
23	Phenylacetaldehyde	1048	0.5	-	0.7	5.5	5.6	2.0	-	0.3	3.2	7.0
24	*trans*-β-Ocymene	1052	-	0.7	0.8	-	0.7	-	2.4	4.1	0.0	-
25	γ-Terpinene	1064	1.1	4.4	2.8	-	0.9	-	6.6	8.1	0.3	-
26	Octan-1-ol	1074	-	-	1.1	-	-	-	0.5	0.5	-	-
27	α-Terpinolene	1092	-	0.4	0.1	-	-	-	0.3	1.1	-	-
28	Nonan-2-one	1094	-	0.4	-	-	-	-	0.8	-	-	-
29	Methyl benzoate	1098	-	0.4	-	0.2	-	-	0.3	-	-	-
30	Linalool	1102	15.2	21.7	21.3	1.2	1.6	3.8	21.1	10.4	5.3	6.8
31	2-Phenylethanol	1118	2.9	4.1	2.9	1.3	2.7	6.7	2.8	1.2	0.6	1.2
32	Methyl octanoate	1128	-	-	-	-	-	-	0.2	0.4	-	-
33	Phenylacetonitrile	1143	11.0	-	1.9	7.2	7.3	10.4	-	1.2	5.0	6.3
34	Isopulegol	1151	-	-	5.2	-	-	-	-	1.8	-	-
35	Isomenthone	1159	-	-	0.3	-	-	-	-	0.0	-	-
36	Nonan-1-ol	1178	-	0.2	-	-	-	-	0.8	-	-	-
37	Terpinen-4-ol	1181	4.1	5.9	5.8	0.3	-	2.0	3.6	1.5	0.9	0.7
38	*p*-Cymen-8-ol	1189	-	-	-	-	-	-	0.2	-	-	-
39	α-Terpineol	1194	10.2	16.0	11.4	3.0	2.4	9.3	9.0	4.1	4.1	6.1
40	2-Aminobenzaldehyde *	1218	-	-	0.1	0.5	1.1	-	0.2	0.1	0.3	-
41	Methyl nonanoate	1228	-	-	-	-	-	-	0.5	0.8	0.6	-
42	Piperitone	1253	-	0.1	0.1	-	-	-	0.1	0.0	0.0	-
43	Geraniol	1260	-	0.5	0.5	-	-	-	0.7	0.8	1.2	-
44	1*H*-Indole	1295	7.9	7.3	11.5	52.5	52.3	8.9	12.2	16.5	47.4	39.6
45	Methyl decanoate	1328	-	-	-	-	-	-	-	0.4	-	-
46	Methyl anthranilate	1344	7.7	6.1	3.9	8.5	3.0	19.8	5.6	5.6	9.1	9.1
47	β-Elemene	1394	-	0.5	-	-	1.1	-	0.9	1.5	-	-
48	*cis*-Jasmone	1399	5.1	3.6	2.8	3.0	1.1	6.9	5.1	6.0	7.9	2.8
49	*trans*-Caryophyllene	1421	-	1.3	0.8	-	2.2	-	1.5	2.6	-	-
50	α-Humulene	1456	-	0.2	0.3	-	-	-	0.5	0.8	-	-
51	(*E*,*Z*)-α-Farnesene	1496	-	-	-	-	-	-	0.5	0.0	-	-
52	(*E*,*E*)-α-Farnesene	1503	-	0.4	0.1	-	-	-	2.0	2.6	-	0.7
53	Methyl dodecanoate	1523	-	-	-	-	-	-	0.2	-	-	0.9
54	Caryophyllene oxide	1584	-	-	-	-	-	-	-	0.7	-	-
55	Methyl tetradecanoate	1727	-	-	0.3	0.5	1.1	-	0.9	2.2	-	1.6
56	Methyl hexadecanoate	1929	-	0.4	0.9	1.3	2.7	-	-	5.5	-	6.1

HoS = honey-sac, NE_A_ = nectar *Kawano Wase*, NE_B_ = nectar *Chahara*, NE_C_ = nectar *Okitsu*, NE_D_ = nectar *Zorica*, RI = retention indices on HP-5MS column, * = tentatively identified.

**Table 2:** The results of GC-FID and GC-MS analysis of the extracts from the honey-sac (HoS) and nectars (NE) obtained by USE.

No.	Compound	RI	Area (%)				
			HoS	NE_A_	NE_B_	NE_C_	NE_D_
1	2-Phenylethanol	1118	-	1.9	-	-	-
2	Phenylacetic acid	1262	3.0	-	-	-	-
3	1*H*-Indole	1295	1.6	6.8	2.7	10.3	6.4
4	Methyl anthranilate	1344	1.4	11.7	0.9	1.4	1.2
5	1-Hydroxylinalool **	1366	5.4	-	-	-	-
6	1,3-Dihydro-2*H*-indol-2-one	1471	6.4	18.1	0.2	3.1	0.8
7	Caffeine	1842	11.5	8.9	1.1	4.3	2.1
8	Hexadecan-1-ol	1882	11.6	6.9	10.7	15.7	16.5
9	Methyl hexadecanoate	1929	-	4.3	-	13.7	0.0
10	(*Z*)-Octadec-9-en-1-ol	2059	45.3	25.4	69.2	14.3	52.3
11	Octadecan-1-ol	2084	8.6	6.1	5.3	26.0	12.7

HoS = honey-sac, NE_A_ = nectar *Kawano Wase*, NE_B_ = nectar *Chahara*, NE_C_ = nectar *Okitsu*, NE_D_ = nectar *Zorica*, RI = retention indices on HP-5MS column, ** = correct isomer is not identified.

**Table 3:** The hedaspace composition of *C. unshiu* honey (*n* = 12) determined by HS-SPME, followed GC-FID and GC-MS analysis.

No.	Compound	RI	Area (%) Fibre PDMS/DVB				Area (%) Fibre DVB/CAR/PDMS			
			Min.	Max.	Av.	SD.	Min.	Max.	Av.	SD.
1	Ethanol	<900	0.0	6.7	2.28	2.57	0.0	1.3	0.26	0.58
2	Acetic acid	<900	0.0	8.1	3.22	3.40	0.0	2.6	0.82	1.03
3	Butanal	<900	0.0	2.3	1.02	1.09	0.0	2.8	0.58	1.24
4	Ethyl acetate	<900	0.0	2.4	0.88	1.21	0.0	0.0	0.00	0.00
5	3-Methylbutanal	<900	0.0	0.9	0.42	0.45	0.0	0.0	0.00	0.00
6	Butan-1-ol	<900	0.0	3.3	1.26	1.73	0.0	0.0	0.00	0.00
7	Pentanal	<900	0.0	0.0	0.00	0.00	0.0	0.7	0.14	0.31
8	3-Hydroxybutan-2-one	<900	0.0	0.0	0.00	0.00	0.0	0.0	0.00	0.00
9	Ethylisocyanide *	<900	0.0	0.3	0.06	0.13	0.0	0.0	0.00	0.00
10	3-Methylbutanenitrile *	<900	0.0	1.4	0.28	0.63	0.0	0.0	0.00	0.00
11	Butanoic acid	<900	0.0	0.7	0.16	0.30	0.0	0.0	0.00	0.00
12	3-Methylbutan-1-ol	<900	0.0	1.9	0.38	0.85	0.0	0.0	0.00	0.00
13	Octane	<900	0.0	1.5	0.46	0.68	0.0	2.7	0.94	1.03
14	Hexanal	<900	0.0	0.7	0.22	0.32	0.0	1.3	0.33	0.65
15	Furfural	<900	0.5	1.9	1.18	0.51	0.0	0.6	0.24	0.33
16	Dihydro-2-methyl-3(2*H*)-furanone	<900	0.0	0.9	0.18	0.40	0.0	0.0	0.00	0.00
17	Isoamylacetate	<900	0.0	1.9	0.38	0.85	0.0	0.0	0.00	0.00
18	Nonane	900	0.0	0.0	0.00	0.00	0.0	1.1	0.24	0.48
19	Heptanal	902	0.0	0.0	0.00	0.00	0.0	0.3	0.10	0.14
20	Benzaldehyde	965	5.8	9.8	7.18	1.86	3.3	6.6	5.06	1.46
21	Hexanoic acid	980	0.0	0.8	0.26	0.37	0.0	0.0	0.00	0.00
22	6-Methylhept-5-en-2-one	989	0.0	0.0	0.00	0.00	0.0	0.9	0.30	0.39
23	Ethyl hexanoate	1001	0.0	0.4	0.08	0.18	0.0	0.0	0.00	0.00
24	Octanal	1004	0.0	0.0	0.00	0.00	0.4	0.9	0.58	0.22
25	*p*-Cymene	1029	0.0	0.0	0.00	0.00	0.0	0.4	0.22	0.16
26	Benzyl alcohol	1038	0.0	2.8	1.32	1.02	0.0	0.0	0.00	0.00
27	Phenylacetaldehyde	1048	34.4	47.2	41.92	5.85	38.3	49.1	43.36	4.35
28	*cis*-Linalool oxide	1075	3.0	11.5	5.48	3.49	0.0	4.1	1.94	1.51
29	*p*-Cymenyl	1095	0.0	0.8	0.36	0.38	0.7	2.7	1.66	0.81
30	Methyl benzoate	1098	0.0	6.2	1.24	2.77	0.0	14.5	2.90	6.48
31	Linalool	1102	0.0	2.2	1.30	0.87	0.0	4.5	3.18	1.83
32	Hotrienol	1105	1.4	2.6	1.96	0.56	1.2	2.3	1.66	0.42
33	Methyl octanoate	1128	2.0	4.9	3.12	1.45	2.3	5.3	3.88	1.38
34	Phenylacetonitrile	1143	2.7	9.9	5.44	3.12	3.4	10.2	6.62	3.04
35	Lilac aldehyde (isomer I) **	1173	1.0	5.6	2.82	2.09	1.5	7.2	3.80	2.69
36	Lilac aldehyde (isomer II) **	1178	0.0	0.5	0.10	0.22	0.0	0.8	0.16	0.36
37	Lilac aldehyde (isomer III) **	1188	0.0	0.0	0.00	0.00	0.0	0.4	0.16	0.22
38	Octanoic acid	1194	0.0	0.0	0.00	0.00	0.0	0.4	0.12	0.18
39	Dill ether	1198	0.0	0.7	0.14	0.31	0.0	0.0	0.00	0.00
40	α-Terpineol	1194	0.0	0.7	0.18	0.30	0.9	3.2	1.58	0.94
41	Decanal	1207	0.0	1.4	0.56	0.59	1.4	5.1	3.46	1.48
42	Methyl nonanoate	1217	0.0	1.0	0.20	0.45	0.0	0.0	0.00	0.00
43	8,9-Epoxy-*p*-menth-1-ene *	1218	0.0	0.0	0.00	0.00	0.0	4.6	2.82	1.80
44	*p*-Meth-9-en-1-al (isomer I) **	1221	0.0	1.1	0.22	0.49	0.0	0.0	0.00	0.00
45	*p*-Meth-9-en-1-al (isomer II) **	1257	0.0	0.5	0.10	0.22	0.0	0.5	0.10	0.22
46	4-Methoxybenzaldehyde	1276	0.0	0.3	0.06	0.13	0.0	0.7	0.22	0.32
47	3-Methyl-6-(1-methylethyl)-cyclohex-2-en-1-one	1258	0.0	0.0	0.00	0.00	0.0	1.2	0.52	0.52
48	Nonanoic acid	1272	2.1	3.3	2.86	0.50	2.3	4.9	3.60	1.25
49	1*H*-Indole	1295	0.0	0.0	0.00	0.00	0.0	0.8	0.40	0.38
50	Methyl anthranilate	1344	0.0	3.3	0.78	1.42	0.0	0.0	0.00	0.00
51	Methyl hexadecanoate	1929	0.0	0.5	0.00	0.22	0.0	0.0	0.00	0.00

Min. = minimal percentage, Max. = maximal percentage, Av. = average percentage, SD. = standard deviation, RI = retention indices on HP-5MS column, * = tentatively identified, ** = correct isomer is not identified.

**Table 4:** The results of GC-FID and GC-MS analysis of *C. unshiu* honey (*n* = 12) ultrasonic solvent extracts.

No.	Compound	RI	Area (%) USE (Pentane/Et_2_O 1:2 (*v*/*v*))				Area (%) USE (CH_2_Cl_2_)			
			Min.	Max.	Av.	SD.	Min.	Max.	Av.	SD.
1	Ethylbenzene	<900	0.1	0.8	0.24	0.31	0.0	0.1	0.03	0.05
2	1,3-Dimethylbenzene **	<900	0.2	3.1	0.88	1.25	0.0	0.1	0.03	0.05
3	1,4-Dimethylbenzene **	<900	0.1	0.8	0.34	0.34	0.0	0.1	0.03	0.05
4	Benzyl alcohol	1038	0.1	0.6	0.22	0.22	0.0	0.1	0.05	0.06
5	Phenylacetaldhyde	1048	0.0	1.2	0.38	0.48	0.0	0.5	0.20	0.24
6	2-Phenylethanol	1118	0.0	0.3	0.12	0.11	0.0	0.1	0.05	0.06
7	Benzoic acid	1166	0.1	0.3	0.14	0.09	0.0	0.2	0.08	0.10
8	Decanal	1207	0.0	0.1	0.02	0.04	0.0	0.0	0.00	0.00
9	2,3-Dihydrobenzofuran	1222	0.0	0.0	0.00	0.00	0.0	0.1	0.05	0.06
10	Phenylacetic acid	1262	0.1	0.3	0.18	0.08	0.1	0.2	0.15	0.06
11	1*H*-Indole	1295	0.1	0.3	0.14	0.09	0.1	2.6	0.90	1.16
12	1-Hydroxylinalool **	1366	0.5	6.5	2.16	2.58	0.2	2.0	1.25	0.79
13	4-Hydroxy-2-phenylethanol	1425	0.1	0.3	0.20	0.07	0.0	0.5	0.25	0.29
14	1,3-Dihydro-2*H*-indol-2-one	1471	0.2	1.7	0.74	0.60	0.1	1.3	0.53	0.57
15	Tetradecan-1-ol	1676	0.5	1.3	0.72	0.33	0.0	0.4	0.10	0.20
16	1*H*-indole-2,3-dione *	1698	0.0	0.1	0.02	0.04	0.0	0.9	0.45	0.37
17	Heptadecane	1700	0.0	1.0	0.30	0.42	0.0	0.0	0.00	0.00
18	Methyl syringate	1744	0.0	0.4	0.10	0.17	0.0	1.3	0.33	0.65
19	4-Hydroxy-3,5,6-trimethyl-4-(3-oxo-1-butenyl)cyclohex-2-en-1-one (Vomifoliol)	1796	0.2	2.0	0.84	0.81	0.2	2.4	1.53	1.04
20	Octadecane	1800	0.0	0.1	0.04	0.05	0.0	0.5	0.13	0.25
21	Caffeine	1842	0.3	0.8	0.56	0.21	1.2	7.1	3.15	2.71
22	4-(1-Methyl-1-phenylethyl)phenol	1858	0.0	0.0	0.00	0.00	0.0	0.2	0.05	0.10
23	Hexadecan-1-ol	1882	5.8	15.3	10.14	3.51	2.4	5.4	3.38	1.38
24	Nonadecane	1900	0.0	1.3	0.26	0.58	0.0	0.0	0.00	0.00
25	Hexadecanoic acid	1963	0.5	6.6	4.02	2.88	7.3	33.8	14.90	12.71
26	Methyl 1*H*-indole-3-acetate	1980	0.0	0.2	0.04	0.09	0.0	5.6	1.95	2.62
27	Eicosane	2000	0.0	0.4	0.12	0.18	0.1	0.9	0.45	0.37
28	2,3-Dihydro-5,7-dihydroxy-2-phenyl-4*H*-1-benzopyran-4-one	2010	0.0	0.0	0.00	0.00	1.0	19.6	8.48	8.22
29	(*Z*)-Octadec-9-en-1-ol	2059	20.5	49.1	36.54	10.88	11.8	22.8	18.73	5.18
30	Octadecan-1-ol	2084	4.5	11.4	7.70	2.97	3.8	5.4	4.80	0.70
31	Heneicosane	2100	0.0	3.7	0.78	1.63	0.0	3.0	0.75	1.50
32	(*Z*)-Octadec-9-enoic acid	2142	0.0	0.0	0.00	0.00	0.0	15.7	5.65	7.28
33	Docosane	2200	0.2	15.7	7.34	7.73	0.0	8.5	3.30	3.76
34	(*Z*)-Tricos-9-ene	2264	0.0	1.6	0.50	0.73	0.0	2.3	0.58	1.15
35	Tricosane	2300	0.3	26.4	7.54	10.93	1.5	8.9	3.80	3.43
36	Tetracosane	2400	1.1	20.8	9.50	8.38	0.1	29.7	16.05	12.75

Min. = minimal percentage, Max. = maximal percentage, Av. = average percentage, SD. = standard deviation, RI = retention indices on HP-5MS column, * = tentatively identified, ** = correct isomer is not identified.
